# Feasibility, Acceptability, and Usability of Physiology and Emotion Monitoring in Adults and Children Using the Novel *Time2Feel* Smartphone Application

**DOI:** 10.3390/s23239470

**Published:** 2023-11-28

**Authors:** Kristel Thomassin, Sadie McVey Neufeld, Nida Ansari, Natasha Vogel

**Affiliations:** Department of Psychology, University of Guelph, Guelph, ON N1G 2W1, Canada; sneufe02@uoguelph.ca (S.M.N.); ansarin@uoguelph.ca (N.A.); vogeln@uoguelph.ca (N.V.)

**Keywords:** emotion, physiology, electrodermal activity, wearable device, experience sampling method

## Abstract

The present study tests the feasibility, acceptability, and utility of the novel smartphone application—*Time2Feel*—to monitor family members’ emotional experiences, at the experiential and physiological level, and their context. To our knowledge, *Time2Feel* is the first of its kind, having the capability to monitor multiple members’ emotional experiences simultaneously and survey users’ emotional experiences when experiencing an increase in physiological arousal. In this study, a total of 44 parents and children used *Time2Feel* along with the Empatica E4 wrist-wearable device for 10 days. Engagement rates were within the acceptable range and consistent with previous work using experience sampling methods. Perceived ease of use and satisfaction fell mostly in the moderate range, with users reporting challenges with connectivity. We further discuss how addressing connectivity would increase acceptability. Finally, *Time2Feel* was successful at identifying physiological deviations in electrodermal activity for parents and children alike, and even though responses to those deviation-generated surveys were largely consistent with random survey responses, some differences were noted for mothers and fathers. We discuss the implications of using *Time2Feel* for understanding families’ emotional and stressful experiences day-to-day.

## 1. Introduction

It is widely accepted that emotional experiences occur on experiential, behavioral, and physiological substrates [1]. Despite this, the majority of laboratory research measuring emotion and emotion regulation has not included these multiple substrates (4.5%) [1]. Furthermore, measuring emotional experiences outside the laboratory setting engenders additional complications, particularly with acquiring physiological data. The *Time2Feel* application (version 1.0) and its pairing with the Empatica E4 wrist-wearable device address this important gap.

Though not as widely used as self-reported data, physiological measures derived from one’s autonomic nervous system can provide insight into the intensity of different emotional states by investigating an individual’s level of arousal in reaction to or response to stimuli around them [2,3,4]. Unlike the recall or social desirability bias that may stem from self-reported data on emotions, physiological measures are not influenced by subjective biases, as they are able to reflect facets of emotional responses that are subconscious in nature [2]. The autonomic nervous system oversees involuntary bodily functions and comprises the sympathetic and parasympathetic nervous systems [5]. The sympathetic nervous system in particular is responsible for the ‘fight or flight’ response and is activated when an individual faces environmental threats or stressors. Electrodermal activity (EDA), a marker of sympathetic activity, is reflective of the electrical conductance on the skin given sweat secretion and increases with sympathetic nervous system activity that arises from stress and strong emotional states [4,6,7]. Being able to survey users about their emotions when the sympathetic nervous system is activated has the potential to yield important information about how users identify and respond to stress and other strong emotional experiences. 

Until recently, it has been quite challenging to measure families’ emotional experiences in their day-to-day lives using multi-method approaches. The closest approximation to this has been the use of experience sampling methods (ESM) [8]. ESM collects real-time information on how individuals feel, act, and think on a momentary basis [9]. ESM acknowledges the importance of context as it relates to collecting representative and meaningful data on the behaviors and feelings of individuals, as assessments are meant to be completed within the context in which they naturally occur [10]. ESM methods have evolved from paper-and-pen formats to other technology-supported formats such as text messages and mobile applications [8,9,10], and such approaches are feasible across child and adult samples [8,10]. Response rates for these methods typically range from 43% to 96% [11,12]. 

Relatively recent advances in wearable technology have allowed ESM to measure individuals’ emotional experiences on a day-to-day basis. Wearables like the Fitbit and Apple Watch allow for the monitoring of various physical and physiological indices, such as skin temperature and heart rate [13,14,15]. Very few devices, however, accurately measure EDA. The Empatica E4 is one such device and is feasible and reliable to use in child and adult populations [16,17,18]. Incorporating the E4 within an ESM design allows for the assessment of physiological markers such as EDA in users’ daily lives. In addition, if paired with a phone application, the app can connect with the wearable device to detect meaningful changes in physiology. This is the basis of our *Time2Feel* app. Furthermore, our aim is to develop an app that can be used by both parents and children, with the ultimate goal of being able to examine family-wide emotion processes.

To our knowledge, *Time2Feel* [19] is the first of its kind, having the capability to monitor multiple members’ emotional experiences simultaneously. Furthermore, the app pioneers the ability to survey users’ emotional experiences when experiencing an increase in physiological arousal. With that said, we acknowledge the fast-growing field of digital biomarkers and remote health monitoring, where wearables have gained significant momentum. The Empatica Embrace 2, as one example, is an FDA-cleared wearable that monitors biomarkers using a proprietary algorithm to detect possible seizures and can send an alert when a seizure is detected. Such advances have not translated to basic science research. Building upon previous work, our *Time2Feel* app monitors family members’ emotional experiences, at both the experiential and physiological levels, on a daily basis. The app randomly prompts users to complete brief surveys on the emotion they are experiencing at that moment, the intensity of that emotion, the person (if any) the user is with, and the activity they are engaging in. The app is paired with the Empatica E4 wristband and monitors users’ electrodermal activity. Significant deviations in EDA, defined as a 15–20% increase in EDA level, trigger notifications for the user to complete an emotion survey. Taken together, *Time2Feel* generates both random and deviation-triggered surveys, allowing users to report on their emotional states at random intervals and during times of physiological arousal.

The present study tests the feasibility, acceptability, and utility of the novel smartphone application—*Time2Feel*—to monitor family members’ emotional experiences, at the experiential and physiological level, and their context. Our pilot testing examined families’ (parents and children) engagement levels with *Time2Feel* over a 10-day period. We examined parents’ and children’s perceived ease-of-use of and satisfaction with *Time2Feel* and whether ease-of-use and satisfaction were correlated with engagement for each family member. To explore the potential utility of *Time2Feel*, we examined the extent to which deviations in physiological arousal were identified and surveyed via the app, i.e., how many deviation surveys family members received, as well as the content of family members’ survey responses to random versus deviation surveys.

## 2. Materials and Methods

### 2.1. Participants

Participants included 22 parents (*M*_age_ = 42.59, *SD* = 6.08) and 22 children (54% boys, *M*_age_ = 10.41, *SD* = 1.97) from 11 families. Families were required to be comprised of two parents and two children, and children were required to be between the ages of 7 and 14 years old, with an age gap of no more than 2 years between the siblings. Parents self-identified as White (55%), followed by Latin American (18%), South Asian (9%), and mixed ethnicities (9%), with two participants who chose not to disclose their ethnicity (9%). Most parents were married (83%) with the remaining being in a common-law relationship (17%), and all parents were biologically related to their children. Parents reported their highest level of education as university (91%) and high school (9%). The median household income reported was over $80,000 per year, with the following income distributions: less than $40,000 (8%) over $80,000 (92%). 

### 2.2. Materials 

#### 2.2.1. *Time2Feel* Phone Application

*Time2Feel* was developed in collaboration with Troon Technologies [20] for both IOS and Android platforms. The app notifies users to complete an emotion survey containing 4 questions (for children) or 5 questions (for parents). The first question asks users “How are you feeling right now?”, where they can select their current emotional state from a list of 13 emotions: happy, nervous, sad, excited, relaxed, frustrated, scared, angry, surprised, proud, ashamed, guilty, and disgusted. Following this selection, users rate the intensity of their chosen emotion on a sliding scale ranging from 0 (very slightly or not at all) to 100 (extremely). The third question asks users “What are you doing right now?” with 12 response options: homework, cooking, chores, shopping, eating, playing games, work, exercising, watching TV, relaxing, social media, and sports/outdoor activities. Finally, users are asked to identify who they are with at that moment and can select from 8–9 options depending on the user (Children: alone, mom, dad, brother, sister, whole family, friend, group of friends; parents: alone, significant other, younger son, older son, younger daughter, older daughter, whole family, friend, group of friends). Finally, parents receive a fifth question asking, “How stressed are you feeling right now about parenting?”, where parents can rate their parenting stress on a sliding scale ranging from 0 (very slightly or not at all) to 100 (extremely). 

We programmed *Time2Feel* to randomly generate survey notifications on a stratified random-interval schedule in which study hours were divided into four equal intervals and a survey notification was sent randomly during each interval. Study hours were limited to 4 p.m. to 8 p.m. on weekdays and 10 a.m. to 6 p.m. on weekends. This was to ensure we did not interfere with school hours. In total, users received four random surveys each day for 10 days. If users missed completing their survey, then one reminder notification was sent within the same interval.

In addition to these random survey notifications, *Time2Feel* also communicates with the Empatica E4 wristband through an application programming interface (API). Although Empatica provides developer tools, it does not allow other apps to sync with their portal, E4 Connect. As a result, our team developed a solution for streaming data to our server. The deviation algorithm is applied to the raw EDA data being streamed and triggers a survey when a deviation is identified, to identify meaningful deviations in EDA. To do this, a baseline EDA reading is obtained from each user at the initial researcher-led orientation to the device and app. The mean EDA level captured during this 3-min calibration is saved and used as the baseline marker for any potential deviation. Our algorithm to determine a ‘meaningful’ deviation was based on benchmarks found in existing research (e.g., [21]). Specifically, previous research has shown increases in EDA levels of around 15% [21]. However, to prevent identifying deviations based on noise or artifacts, we limited the increase in arousal to 20%. EDA is measured in microsiemens (µS), with ranges typically within the 0–2 µS range in the absence of stimuli [22]. To not overwhelm participants with survey notifications, we limited the generation of deviation-triggered survey notifications to four per day (similar to the random survey notifications). Participants did not know if they were receiving a random or deviation-generated survey. For both random and deviation surveys, users had 15 min to complete the survey following the notification prompt.

#### 2.2.2. Empatica E4 Wristband

Each family member was fitted with an Empatica E4 wristband [23], which collected real-time continuous EDA at a sampling frequency of 4 Hz (i.e., four samples per second) throughout the allotted study period. The Empatica E4 wristband is a validated tool for collecting EDA in both adult and youth samples [16,17,18,24]. Each wristband contains four sensors: EDA, photoplethysmography, infrared thermophile, and a 3-axis accelerometer. EDA measurement is based on two electrodes attached to the end of the wristband. Once attached, the electrodes sit against the inside of the wrist, and the current passing between the two electrodes captures fluctuating changes in the electrical properties of the skin as measured in microsiemens (μS). Though the wrist is not considered a ‘gold-standard’ location to measure EDA, as there is a lower density of sweat glands compared to other sites (i.e., palmar surface) [25], it is much more practical and comfortable for the user and arguably provides an ideal signal-to-noise ratio when considering how often people use their hands in everyday activities [16,26]. The wristband was worn on the participants’ non-dominant hands, snugly, to minimize motion artifacts. Each wristband was connected to the participants’ *Time2Feel* application via Bluetooth.

#### 2.2.3. *Time2Feel* Engagement

Participant engagement levels were used as a proxy for determining the feasibility of *Time2Feel* and were measured by calculating the percentage of *Time2Feel* surveys completed out of the total number of survey prompts received over the course of the 10-day period. A complete response was defined as a survey that had all questions answered, and an incomplete response was defined as a survey that was started but with some answers missing. A separate response rate, reported as a percentage, was calculated for both random and deviation surveys.

#### 2.2.4. Perceived Ease of Use and Satisfaction with *Time2Feel*

Perceived ease-of-use of and satisfaction with *Time2Feel* were measured using participants’ scores on the ease-of-use and satisfaction subscales of the Usefulness, Satisfaction, and Ease-of-use questionnaire (USE) [27]. The USE is a 30-item measure using a 7-point Likert scale, with values ranging from 1 (strongly disagree) to 7 (strongly agree). The satisfaction subscale is comprised of four items (e.g., “I would recommend it to a friend”). Although the ease-of-use subscale contains 11 items (e.g., “Using it is effortless”), we did not include items 7–8, as these items have shown poor factor loadings (≤.20) [28]. Both subscales, across family members, displayed adequate internal consistency (αs .63–.96). At the end of the USE, we also presented family members with an open-ended question, “Please provide any other experiences/thoughts regarding the *Time2Feel* app that you would like us to know”. Text responses to this question were examined qualitatively.

### 2.3. Procedure

To be eligible, families were required to be fluent in reading and speaking English, as all scales and experimental tasks were validated using English-speaking samples. Participants were excluded if they had any impairments with speech, hearing, or sensory integration, had any developmental delays, or had a history of cardiovascular health problems. Eligible families first completed the online demographic questionnaire and then were provided with an equipment package containing four Empatica E4 devices, cell phones for family members who did not have their own (all children used a lab cell phone), and all necessary charging accessories. Following equipment drop-off, participants met with a research assistant on a videoconference call hosted on Microsoft Teams, where they were provided with instructions for the 10-day study period and were oriented to the equipment and the *Time2Feel* application. During this orientation call, each family member, one at a time, was instructed to wear the wristband and provide a baseline EDA reading for three minutes. Families were instructed to wear the wristband and respond to surveys on the *Time2Feel* app over the course of the next 10 days following the orientation session. As mentioned previously, study hours were restricted to 4 p.m. to 8 p.m. on weekdays and 10 a.m. to 6 p.m. on weekends. After the 10-day study period, the equipment was picked up by a research assistant, and family members were instructed to complete the USE questionnaire to document their experiences using the *Time2Feel* application. At the end of each study component (i.e., questionnaires, orientation call, 10-day emotion reporting), each family member received monetary compensation in the form of gift cards, totaling up to $75 for each parent and $25 for each child. The research was approved by the sponsoring institution’s Research Ethics Board (#19-07-018).

### 2.4. Analytic Approach

Data cleaning and restructuring were completed with RStudio [29], and statistical analyses were conducted with SPSS Version 28. One-way random intraclass correlations (ICC) were conducted with *Time2Feel* engagement levels, satisfaction ratings, and ease-of-use ratings to determine whether the aggregation of child data (i.e., the creation of a mean child value for focus children and siblings) was warranted. The ICC is commonly used as an agreement and reliability index for multi-level analyses [30,31,32]. In accordance with LeBreton and Senter’s [32] standards, an ICC(1) value of .00–.30 indicates a lack of agreement, a value of .31–.50 indicates weak agreement, a value of .51–.70 indicates moderate agreement, a value of .71–.90 indicates strong agreement and a value of .91–1.00 indicates very strong agreement between individuals. In the present study, we used an a priori cutoff of .51 to determine that between-family variation was bigger than within-child variation and thus would warrant aggregation. Mothers and fathers were analyzed separately because of existing research showing mother and father differences in their daily emotion-reporting patterns [33].

To examine engagement levels with the *Time2Feel* app, descriptive statistics were inspected across family member types for both random and deviation-generated survey responses. Paired sample *t*-tests were conducted to compare mean engagement levels between parents and children. Cohen’s *d* was reported as a measure of effect size. Cohen’s [34] benchmarks were used as interpretive guidelines, such that an effect size of *d* = 0.20 was considered small, *d* = 0.50 was medium, and *d* = 0.80 was considered large. To explore perceived ease of use and satisfaction among family members, descriptive statistics on scores from the USE subscales were examined for each family member. Paired sample *t*-tests were conducted to compare mean ease-of-use and satisfaction scores between parents and children, again using aggregated values where applicable. 

Finally, to explore the potential utility of *Time2Feel*, we examined the extent to which deviations in EDA were detected. We also examined the descriptive statistics of survey responses to random and deviation surveys, including responses to the emotions and situations reported in each type of survey.

## 3. Results

### 3.1. Descriptive and Preliminary Analyses

ICCs indicated that focus children and siblings were similar in their response rates to random surveys, *ICC*(1) = 0.93, and deviation-generated surveys, *ICC*(1) = 0.56. ICCs were also conducted for satisfaction and ease-of-use scores. Focus children and siblings were similar in their ease-of-use scores, *ICC*(1) = 0.96, and satisfaction levels, *ICC*(1) = 0.87. Focus child and sibling data were thus aggregated for all analyses. Table 1 presents the mean, standard deviation, range, and sample size of each study variable, while Table 2 presents the Pearson correlations between all study variables.

### 3.2. Engagement with Time2Feel

Mean engagement across all family members was 65.94%, with children exhibiting lower rates and a much broader range (*M* = 52.23%, range 3.57–81.50%) compared to parents (*M* = 72.80%, range 45.83–88.71%).

Response rates for random surveys ranged from 3.57% to 95% (*n* = 33, *M* = 64.04, *SD* = 23.18). A paired sample *t*-test indicated that mothers had a higher response rate than fathers, *t*(10) = 2.46, *p* = .034, *d* = 0.74, and children, *t*(10) = 5.09, *p* < .001, *d* = 1.53. Similarly to mothers, fathers also had a significantly higher response rate for random surveys compared to children, *t*(10) = 3.04, *p* = .012, *d* = 0.92. All the effects were large.

For deviation-generated surveys, response rates ranged from 0% to 100% (*n* = 28, *M* = 71.98, *SD* = 20.19). A paired sample *t*-test indicated that mothers had a higher response rate for deviation surveys than fathers, *t*(6) = 2.79, *p* = .032, *d* = 1.05. Regarding parent-child comparisons, there were no significant differences in engagement levels between mothers and children, *t*(7) = 1.02, *p* = .343, *d* = 0.36, or between fathers and children, *t*(8) = 2.13, *p* = .066, *d* = 0.71.

Individual family member responses and associated response rates for random and deviation surveys are included as supplemental material (see Tables S1 and S2).

### 3.3. Perceived Ease of Use and Satisfaction with Time2Feel

Mean ease-of-use scores indicated that overall, family members “somewhat agree” that the *Time2Feel* application is easy to use. Mothers and fathers did not differ in their *Time2Feel* ease-of-use ratings, *t*(10) = 0.24, *p* = .816, *d* = 0.07. Likewise, mothers and children did not endorse different ease-of-use ratings, *t*(10) = 1.76, *p* = .110, *d* = 0.53. However, children did endorse higher ease-of-use with *Time2Feel* than fathers with a medium effect size, *t*(10) = 2.25, *p* = .048, *d* = 0.68. 

Mean satisfaction scores indicated that overall, family members were somewhat satisfied with *Time2Feel*. Mothers and fathers did not differ in their *Time2Feel* satisfaction ratings, *t*(10) = 1.58, *p* = .145, *d* = 0.48. Similarly to the ease-of-use results, children and mothers did not endorse different satisfaction ratings, *t*(10) = 1.37, *p* = .200, *d* = 0.41, but children endorsed significantly higher satisfaction with *Time2Feel* compared to fathers, *t*(10) = 2.83, *p* = .018, *d* = 0.85. 

Ease of use and satisfaction ratings were not significantly correlated with rates of engagement with *Time2Feel*.

We also asked family members to openly share their opinions about *Time2Feel*. Of the 44 family members, 7 mothers, 6 fathers, and 4 children chose to answer this question, producing 22 total comments. Of these, 15 were related to technological problems and wifi access: “There were some glitches with the app. It disconnects regularly. Because it required the Internet, it was difficult to fill out the surveys. If there was an offline way to use it and then it uploaded once the Internet was available, that would be helpful”. Seven comments suggested adding more situation (*n* = 6) and emotion (*n* = 1) response options: “I think there could be more options for ‘what we are doing right now’ when answering a survey. At least put an ‘other’ option”.

### 3.4. Potential Utility of Time2Feel

Over the course of 10 days, nine of the eleven participating mothers received a sum of 160 deviation surveys. The average daily number of deviation-generated surveys received per mother (*n* = 9) was 1.70 (*SD_daily_* = 1.28). Nine fathers received and reported on deviation-generated surveys and received a sum of 189 deviation surveys (*M_daily_* = 2.06, *SD_daily_* = 1.09). Finally, twenty children received an overall sum of 405 deviation surveys (*M_daily_* = 2.08, *SD_daily_* = 1.07).

Of the 13 emotion options provided, mothers and fathers most frequently endorsed happy (36–39%), content (30–34%), frustrated (12–13%), and nervous (7–10%) in response to random surveys. Children endorsed feeling content (34%), happy (32%), excited (11%), and frustrated (10%). See Table 3.

In response to deviation-generated surveys, mothers endorsed happy (31%), content (17%), and frustrated (13%). Paired-sample *t*-tests examined whether emotions endorsed on random versus deviation surveys significantly differed. Results indicated that mothers endorsed ‘content’ with a higher frequency (trend level) in response to random (versus deviation-generated) surveys, *t*(10) = 2.10, *p* = .062, *d* = .64. The highest endorsed emotions reported by fathers followed the same pattern as those for mothers. Fathers’ endorsement of ‘content’ in response to random (versus deviation-generated) did not statistically differ, though the size of the difference was small-to-moderate, *t*(10) = 0.93, *p* = .377, *d* = .39. In terms of mother and father differences, a large effect was noted for fathers’ versus mothers’ endorsement of ‘proud’ in response to random surveys, though this did not reach statistical significance, *t*(10) = 1.83, *p* = .097, *d* = .86. Similarly to their reports on the random surveys, children reported mostly feeling content (34%), happy (26%), excited (14%), and frustrated (11%). See Table 3.

Of the 12 situation options provided, the most endorsed situations reported by mothers in response to random surveys included doing chores, relaxing, work, and eating. The main situations reported by fathers included working, relaxing, watching TV, and eating. Children highly endorsed relaxing, playing games, watching TV, and eating. See Table 4.

In response to deviation-generated surveys, mothers endorsed doing chores, relaxing, work, watching TV, and eating. Situations endorsed by fathers included work, relaxing, playing games, chores, and watching TV. Children mainly endorsed relaxing, watching TV, playing games, and eating. The primary difference between random and deviation-generated surveys was with mothers endorsing ‘chores’ and fathers endorsing ‘playing games’ and ‘chores’ to a greater degree in response to deviation (versus random) surveys. See Table 4.

## 4. Discussion

This study introduces the *Time2Feel* smartphone application as a novel method to capture users’ emotional experiences, at both experiential and physiological levels, within naturalistic contexts. Specifically, *Time2Feel* employs an ESM approach, coupled with wrist-wearable technology, to measure families’ emotional experiences in a stratified random interval schedule (time-based sampling) and when physiological arousal is detected (event-based sampling). We examined the potential utility of *Time2Feel* by exploring families’ app engagement, perceived ease-of-use and satisfaction with the app, and responses to deviation-triggered prompts.

Across all family members, engagement with *Time2Feel* was 66%. Due to previously reported differences in ESM engagement between adult and child populations [12], it is important to evaluate parents’ and children’s response rates separately. Parents’ engagement was consistent with, or even slightly higher than average rates and ranges reported in recent systematic reviews surveying mobile-based ESM designs in adults (*M* = 71.6%, [11]; *M* = 64.7%, [35]). In addition, previous studies have reported response rates as low as 43 and 31%, which our reported range far exceeds [36]. This indicates that *Time2Feel* elicits effective and positive engagement in adults. Amongst children, while the average response rate was significantly lower than the reported rate in a systematic review of children and adolescents’ ESM engagement (78.3%, [12]), studies exploring ESM engagement in middle childhood specifically (ages 7–12) have reported relatively lower rates. For example, studies using time-based sampling protocols (i.e., random prompts within a set timeframe) with children in middle childhood have reported response rates ranging from 60–69% [37,38]. Furthermore, ESM studies employing event-based sampling protocols (i.e., prompts triggered in response to an event, such as an increase in physical activity) have reported youth engagement levels as low as 47.9% [39]. Furthermore, there were three child users who barely engaged with *Time2Feel*. It should be noted that these users greatly affected mean engagement levels. Taken together, it is notable that we were able to achieve engagement levels at an average minimum threshold of 52% given the age of our child participants and the added complexity of the Empatica E4 device and associated deviation-triggered prompts. Indeed, *Time2Feel* is unique in its integrated time-based and event-based sampling approach that relies on the ability to detect deviations from individual EDA baselines. 

Distinct family members displayed different levels of engagement with *Time2Feel.* Specifically, mothers had the highest response rate across families compared to fathers (for both random and deviation prompts) and children (for random prompts), and children had the lowest random response rate compared to both mothers and fathers. Higher engagement from mothers in this sample is unsurprising, as all mothers initiated their family’s participation in the study. As such, mothers may have been particularly inclined and interested to engage with *Time2Feel* and the research process generally compared to their family members. Furthermore, research suggests fathers have traditionally been less engaged in research than mothers [40]. Indeed, fathers and their unique contributions have been historically neglected in child and family research in comparison to mothers [41,42,43], which may result in fathers feeling undervalued in the research process [40,44]. 

There are several potential reasons for children’s lower levels of engagement compared to parents. First, children in our sample were as young as seven years old, and many did not possess their own mobile phones at the time of recruitment. As such, many children may not have been in the habit of regularly using or engaging with a mobile device. For instance, they may forget to check their phone or mistakenly leave it in a separate area of their home, thereby ‘missing’ *Time2Feel* prompts. Next, children may be particularly prone to waning motivation and consistent engagement with repetitive ESM prompts over extended periods of time [12,38,45,46]. Finally, many families reported that children were engaged in various extracurricular activities on evenings and weekends, which may have further impeded children’s ability to consistently engage with *Time2Feel.*

In sum, adults’ engagement levels with *Time2Feel* are consistent with previously reported rates, while children’s engagement levels are somewhat lower than rates reported in similar populations. Despite this, hindrances to children’s engagement may not be unique to *Time2Feel* but rather reflect common ESM challenges with youth, such as lower engagement with event-based prompting or waning motivation over time. Along with the unique physiological event-based prompting capabilities of *Time2Feel*, these findings suggest that *Time2Feel* shows promise as a feasible research tool that participants can effectively engage with in their daily lives. Future research may benefit from adjusting study hours so as to generate prompts that do not interfere with extracurricular activities.

Family members endorsed that they “somewhat agree” that *Time2Feel* is easy to use, and they were “somewhat” that satisfied with the application. While these ratings reflect neutral perceptions of *Time2Feel*, many participants’ qualitative comments reflected specific issues with interrupted Bluetooth connections between *Time2Feel* and the Empatica E4, as well as the need for ‘offline’ capabilities (i.e., the ability to use the application outside of wireless Internet zones). As such, it is expected that participants’ subjective perceptions of *Time2Feel* will become more positive as the research and development team work to improve this distinct pairing feature and explore possibilities for offline access. Surprisingly, while children exhibited the lowest *Time2Feel* engagement levels within families, they endorsed the highest ease-of-use and satisfaction levels regarding the application. Children displayed significantly more positive perceptions of *Time2Feel* compared to fathers, but not mothers. It is possible that children especially enjoyed the novelty of using a mobile application regularly, particularly the large proportion of children who did not previously possess a personal mobile phone. 

Across participants, perceived ease-of-use and satisfaction were not correlated with *Time2Feel* engagement. Factors such as interruptions to Bluetooth connectivity or families’ busy schedules may have primarily impacted families’ abilities to engage with *Time2Feel*, but not their experiences of ease or satisfaction. As children displayed the lowest engagement with *Time2Feel* but the most positive perceptions, this may suggest that participants’ perceptions of ease or satisfaction may not hinder or hold importance for participant engagement. In addition, researchers may consider addressing ongoing improvements to the feasibility and acceptability of *Time2Feel* as conceptually separate endeavors. 

Regarding the usability of *Time2Feel,* family members received approximately two deviation-triggered prompts per day in addition to the four random prompts. This suggests that *Time2Feel* consistently and effectively detects instances of heightened physiological arousal, as indexed by EDA, in participants of various ages. Even though emotions and situations reported via the random and deviation-generated surveys were largely similar and no statistically significant differences emerged, it is worth noting there were some trend-level differences with effect sizes ranging from small to large. Given that this is a pilot study, these results suggest that further testing is warranted, as *Time2Feel* could have the potential to detect unique emotional experiences when paired with a wearable device. While notable, this finding should be interpreted with caution, considering the possibility that some recorded deviations in physiology may occasionally result from ‘noise’ due to participant movement or other environmental encounters, rather than reflecting genuine changes in participants’ physiological and/or emotional experience. With that said, we did employ a strict deviation algorithm that would omit some of this noise.

There are several limitations to this study that warrant discussion. First, our sample size is small, and thus we present only preliminary findings regarding the feasibility of *Time2Feel*. While our results provide support for the research utility of *Time2Feel* and offer opportunities for continued development and improvement, they should be interpreted with reasonable caution until findings are replicated with more robust sample sizes. We did not measure whether deviations in EDA were subjectively experienced as moments of stress by the participants. Future research may wish to do so. Next, technology issues were noted by participants. For instance, participants reported experiencing disconnected *Time2Feel*-E4 pairings due to physical distance between each device (e.g., each device in different rooms of the same house), being outside of a wireless Internet zone or without a mobile data plan, or sporadic connectivity issues when attempting to pair. Despite these challenges, family members completed, on average, about 4 prompts per day for 10 days, totaling roughly 38–40 surveys per individual. Furthermore, excessive movement with the E4 may have resulted in noise artifacts, although we attempted to account for this by implementing an upper limit to the deviation algorithm. Finally, the daily timeframes for receiving generated prompts (4–8 pm on weeknights, 10–6 pm on weekends) may have coincided with families’ extracurricular activities and outings, which could have limited participant availability to complete *Time2Feel* prompts. This timeframe likely also limited our ability to obtain surveys across a wide range of daily activities.

## 5. Conclusions

This study presents preliminary feasibility, acceptability, and usability data for the novel emotion-tracking smartphone application, *Time2Feel*. *Time2Feel* provides an unprecedented approach to simultaneously capturing both participants’ subjective emotional and objective physiological experiences within their natural environment. Specifically, *Time2Feel* employs an integrated time-based (random prompting) and event-based (deviation prompting) ESM method to comprehensively capture parents’ and children’s emotional experiences. While mothers displayed the highest engagement levels with *Time2Feel* compared to fathers and children, children endorsed the most positive ratings of the application, especially when compared to fathers. Preliminary data suggest *Time2Feel* could hold potential for the detection of unique emotional experiences. Future studies may consider approaches to affirm *Time2Feel* as consistently attractive and engaging for the entire family unit across extended timeframes. In addition, ways of reducing potential barriers to participant engagement, such as addressing Bluetooth connectivity issues and providing consistent access to *Time2Feel* regardless of wireless Internet availability, should be prioritized in future application development. Nonetheless, this study builds upon a burgeoning body of work on digital biomarkers and remote health monitoring using wearable technologies [47,48,49,50,51].

## Figures and Tables

**Table 1:** Means, standard deviations, ranges, and sample sizes of study variables.

	Mean (SD)	Range	*N*
1. Child Age (Months)	132.27 (19.23)	104–162	11 (pairs)
Random Response Rate (%)			
2. Mothers	79.62 (9.23)	65.00–95.00	11
3. Fathers	67.92 (15.68)	44.19–90.00	11
4. Children	44.60 (26.26)	3.57–81.25	11 (pairs)
Deviation Response Rate (%)			
5. Mothers	74.18 (29.21)	0.00–100.00	9
6. Fathers	64.64 (13.78)	42.86–82.61	9
7. Children	76.61 (14.60)	50.00–93.07	10 (pairs)
Ease of Use			
8. Mothers	4.40 (0.83)	3.25–6.00	11
9. Fathers	4.35 (0.93)	2.50–5.75	11
10. Children	4.91 (1.01)	3.06–5.94	11 (pairs)
Satisfaction			
11. Mothers	3.93 (1.67)	1.50–6.00	11
12. Fathers	3.43 (1.21)	1.50–5.50	11
13. Children	4.74 (1.09)	3.25–6.75	11 (pairs)

**Table 2:** Correlations between child age, random and deviation response rates, and ease of use and satisfaction ratings.

	2	3	4	5	6	7	8	9	10	11	12	13
1. Child Age	−.43	−.29	.08	−.01	−.61 ^†^	−.48	−.07	.02	−.47	.26	.16	−.58 ^ † ^
Random Survey Response Rate												
2. Mother Rdm %	-	.28	.52 ^ † ^	.46	.48	.19	.14	.24	.51	−.09	−.31	.21
3. Father Rdm %		-	.35	−.31	.46	.72 *	−.49	−.22	.10	−.32	−.18	.19
4. Child Rdm %			-	.44	−.05	.57 ^ † ^	−.02	.29	.08	.02	−.01	−.31
Deviation Survey Response Rate												
5. Mother Dev %				-	−.29	−.67 ^ † ^	.04	.02	.06	−.43	−.71 *	−.19
6. Father Dev %					-	.33	−.60 ^ † ^	−.20	.44	−.52 ^ † ^	−.46	.37
7. Child Dev %						-	−.32	−.23	−.00	−.18	−.05	−.02
Ease of Use of *Time2Feel*												
8. Mother EOU							-	.75 **	.48	.62 *	.58 ^ † ^	.40
9. Father EOU								-	.65 *	.45	.65 *	.36
10. Child EOU									-	−0.03	.15	.76 **
Satisfaction with *Time2Feel*												
11. Mother SAT										-	.78 **	.05
12. Father SAT											-	.12
13. Child SAT												-

Note. EOU = Ease-of-Use Subscale. SAT = Satisfaction Subscale. * *p* < 0.05; ** *p* < 0.01, † is indicative of a non-significant correlation greater than 0.50.

**Table 3:** Emotions endorsed by each family member in response to random and deviation surveys across 10 days.

Emotion	Mothers		Fathers		Children	
	Random	Deviation	Random	Deviation	Random	Deviation
Happy	39.23%	31.26%	35.64%	45.00%	31.57%	26.28%
Nervous	10.14%	5.51%	6.71%	7.50%	4.80%	5.44%
Sad	3.04%	4.86%	1.15%	-	3.03%	4.23%
Excited	4.15%	0.73%	3.34%	3.33%	10.61%	13.90%
Content	30.31%	16.87%	34.06%	27.50%	34.34%	33.53%
Frustrated	11.64%	13.21%	12.93%	14.17%	9.60%	10.88%
Afraid/Scared	-	-	0.32%	0.83%	1.52%	1.81%
Angry	0.63%	-	1.55%	0.83%	1.26%	1.51%
Surprised	0.30%	0.28%	-	0.83%	0.76%	0.60%
Proud	0.56%	-	3.10%	-	1.01%	0.91%
Ashamed	-	-	-	-	0.76%	0.30%
Guilty	-	-	0.79%	-	0.25%	0.60%
Disgusted	-	-	0.40%	-	0.51%	-

**Table 4:** Situations endorsed by each family member in response to random and deviation surveys across 10 days.

Emotion	Mothers		Fathers		Children	
	Random	Deviation	Random	Deviation	Random	Deviation
Homework	1.39%	-	0.68%	1.31%	3.80%	3.63%
Cooking	5.73%	1.47%	5.03%	1.90%	1.46%	1.60%
Chores	23.91%	21.70%	7.77%	7.31%	7.57%	5.20%
Shopping	5.63%	0.76%	6.73%	3.00%	0.28%	1.58%
Eating	11.69%	10.81%	10.17%	9.76%	9.40%	7.42%
Playing Games	2.24%	1.76%	5.60%	12.71%	20.33%	13.97%
Work	11.42%	16.43%	26.59%	23.59%	-	-
Exercising	2.78%	2.46%	0.28%	-	5.45%	6.91%
Watching TV	9.13%	18.43%	10.31%	14.87%	16.79%	23.50%
Relaxing	18.95%	20.35%	17.91%	13.10%	21.64%	28.13%
Social Media	2.82%	3.41%	1.77%	5.82%	4.09%	5.42%
Sport/Outdoor Activity	4.30%	2.41%	7.15%	6.61%	9.18%	2.11%

## Data Availability

Data for this manuscript are available at https://osf.io/qyjkx/.

## Supplementary Materials

The following supporting information can be downloaded at: https://www.mdpi.com/article/10.3390/s23239470/s1, Table S1: Breakdown Of Response Rates for Random Surveys by Family Member for All Families; Table S2: Breakdown Of Response Rates for Deviation Surveys by Family Member for All Families.
