# Supplementary material for: Feasibility, Acceptability, and Usability of Physiology and Emotion Monitoring in Adults and Children Using the Novel Time2Feel Smartphone Application
Source: Sensors (Basel). 2023 Nov 28;23(23):9470. doi: 10.3390/s23239470 (PMC10708754; doi:10.3390/s23239470)
Supplement: Supplementary file 1 [file sensors-23-09470-s001.zip › sensors-2683178-supplementary.pdf]

Table S1

Breakdown Of Response Rates for Random Surveys by Family Member for All Families

Family ID		# of Initial Surveys Completed	# of Survey Reminders Completed	Total Surveys Completed	Total Surveys Generated	Response Rate (%)
ID001						
	Mother	33	5	38	40	95.00
	Father	32	4	36	40	90.00
	Child	28	4	32	40	80.00
	Sibling	20	6	26	40	65.00
ID002						
	Mother	27	6	33	40	82.50
	Father	18	5	23	40	57.50
	Child	12	5	17	40	42.50
	Sibling	3	4	7	40	17.50
ID003						
	Mother	26	6	32	40	80.00
	Father	26	2	28	40	70.00
	Child	22	4	26	40	65.00
	Sibling	27	3	30	40	75.00
ID004[a]						
	Mother	21	14	35	40	87.50
	Father	30	6	36	40	90.00
	Child	10	3	13	39	33.33
	Sibling	12	3	15	39	38.46
ID005[a]						
	Mother	31	9	40	45	88.89
	Father	9	6	15	32	46.88
	Child	11	5	16	45	35.56
	Sibling	14	3	17	42	40.48
ID006						
	Mother	21	5	26	40	65.00
	Father	18	5	23	40	57.50
	Child	10	4	14	40	35.00
	Sibling	10	3	13	40	32.50
ID007						
	Mother	25	8	33	40	82.50
	Father	19	6	25	40	62.50
	Child	13	8	21	40	52.50
	Sibling	25	8	33	40	82.50

ID008						
	Mother	25	7	32	40	80.00
	Father	22	7	29	40	72.50
	Child	25	5	30	40	75.00
	Sibling	30	5	35	40	87.50
ID009[a]						
	Mother	21	7	28	42	66.67
	Father	11	12	33	42	78.57
	Child	0	1	1	42	2.38
	Sibling	1	1	2	42	4.76
ID011						
	Mother	24	6	30	40	75.00
	Father	28	3	31	40	77.50
	Child	10	9	19	40	47.50
	Sibling	11	11	22	40	55.00
ID012[a]						
	Mother	26	6	32	44	72.73
	Father	13	6	19	43	44.17
	Child	5	0	5	44	11.36
	Sibling	1	0	1	44	2.27

[a]Participants may have received an abnormal number of random surveys (i.e., not 40) either due to coincidences with app testing and troubleshooting, thus receiving more surveys than usual; or, due to prematurely logging out of the application, therefore limiting how many surveys were completed.

Table S2

Breakdown Of Response Rates for Deviation Surveys by Family Member for All Families

Family ID	Total Surveys Completed	Total Surveys Generated	Response Rate (%)
ID001			
Mother	17	22	77.27
Father	19	23	82.61
Child	20	21	95.24
Sibling	10	11	90.91
ID002			
Mother	22	27	81.48
Father	14	21	66.67
Child	19	24	79.17
Sibling	4	12	33.33
ID003			
Mother	--	--	N/A
Father	9	11	81.82
Child	23	31	74.19
Sibling	26	28	92.86
ID004			
Mother	21	25	84.00
Father	20	26	76.92
Child	23	26	88.46
Sibling	20	24	83.33
ID005			
Mother	20	24	83.33
Father	--	--	N/A
Child	24	28	85.71
Sibling	19	29	65.52
ID006			
Mother	6	9	66.67
Father	21	35	60.00
Child	12	13	92.31
Sibling	9	11	81.82
ID007			
Mother	33	37	89.19
Father	17	34	50.00
Child	18	35	51.43
Sibling	22	28	78.57
ID008			
Mother	--	--	N/A

Father	3	7	42.86
Child	9	11	81.82
Sibling	10	12	83.33
ID009			
Mother	0	1	0.00
Father	--	--	N/A
Child	--	--	N/A
Sibling	--	--	N/A
ID011			
Mother	6	7	85.71
Father	14	23	60.87
Child	32	38	84.21
Sibling	27	30	90.00
ID012			
Mother	1	1	100.00
Father	3	5	60.00
Child	1	2	50.00
Sibling	1	2	50.00

Note. Any participants with a response rate of "N/A" were not able to establish a connection between their mobile device and the E4 wristband, either due to the phone or due to a non-functioning wristband.
